# Supplementary material for: Short- and Long-Term Endothelial Inflammation Have Distinct Effects and Overlap with Signatures of Cellular Senescence
Source: Cells. 2025 May 30;14(11):806. doi: 10.3390/cells14110806 (PMC12153915; doi:10.3390/cells14110806)
Supplement: Supplementary file 1 [file cells-14-00806-s001.zip › Supplementary Information_revised.pdf]

Supplementary Information for:

Short- and long-term endothelial inflammation have distinct effects and overlap with signatures of cellular senescence

Barbora Belakova, José Basílio, Manuel Campos-Medina, Anna F. P. Sommer, Ulrike Resch, Johannes Breuss and Johannes A. Schmid

Supplementary Methods:

Suppl. Table S1: Primers used for quantitative reverse-transcription PCR:

**Target mRNA**	**forward primer**	**reverse primer**
TNFα	5'-ACC CTC TCT CCC CTG GAA AGG ACA-3'	5'-TGA GGA ACA AGC ACC GCC TGG A-3'
N-cadherin	5'- CCT CCA GAG TTT ACT GCC ATG AC-3´	5'- GTA GGA TCT CCG CCA CTG ATT C-3´
vimentin	5'-AGG CAA AGC AGG AGT CCA CTG A-3´	5'- ATC TGG CGT TCC AGG GAC TCA T-3´
fibronectin	5'-ACA ACA CCG AGG TGA CTG AGA C-3´	5'-GGA CAC AAC GAT GCT TCC TGA G-3'
GUSB (housekeeping)	5'-AAA CGA TTG CAG GGT TTC AC-3'	5'-TAT TCC CCA GCA CTC TCG TC-3'

Supplementary Figure S1: Genes that were significantly differentially regulated under the three conditions of interest (5h TNFα treatment, 9d TNFα treatment and replicative senescence) were uploaded (for all 3 conditions separately) to the *NetworkAnalyst* web-platform [1] and compared to the protein-protein interactome of the STRING database (using a cut-off of 900 and experimental validation). A first-order network was computed (for 5h and 9d TNFα treatment) and a zero-order network for senescence (due to the high number of altered genes) – and the minimum networks were determined that link all the seed genes (the differentially regulated genes). These minimum networks were downloaded in graphml-format and imported as network files into the Cytoscape software [2] and marked with the respective condition. The Cytoscape STRING app [3] was applied to the networks to allow functional enrichment analysis. All three networks were merged and the intersection of them was determined using the Cytoscape merge function and designated as common. Using the condition marks, the three networks and their common intersection were dragged from each other and thereby visually separated.

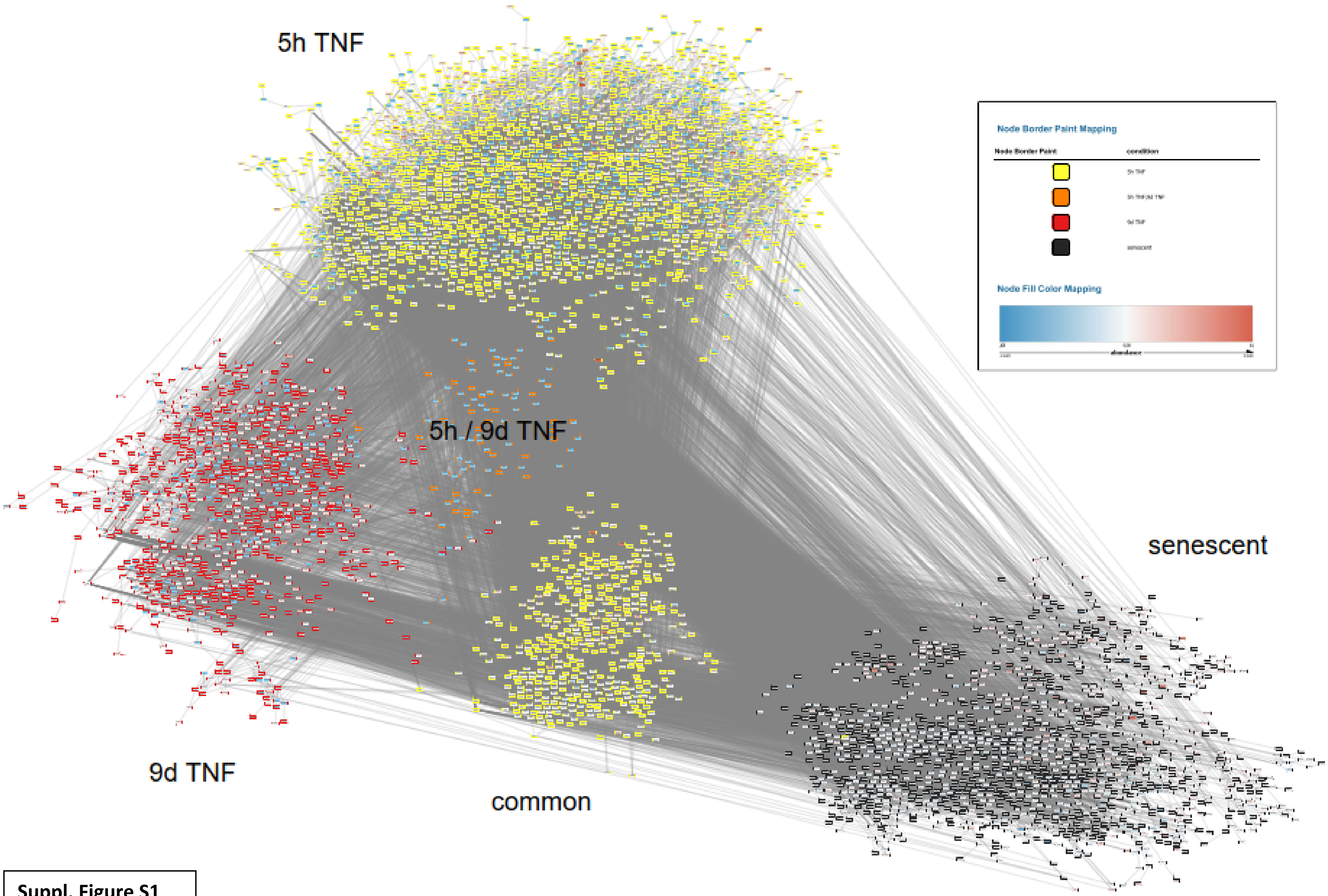

Suppl. Figure S1

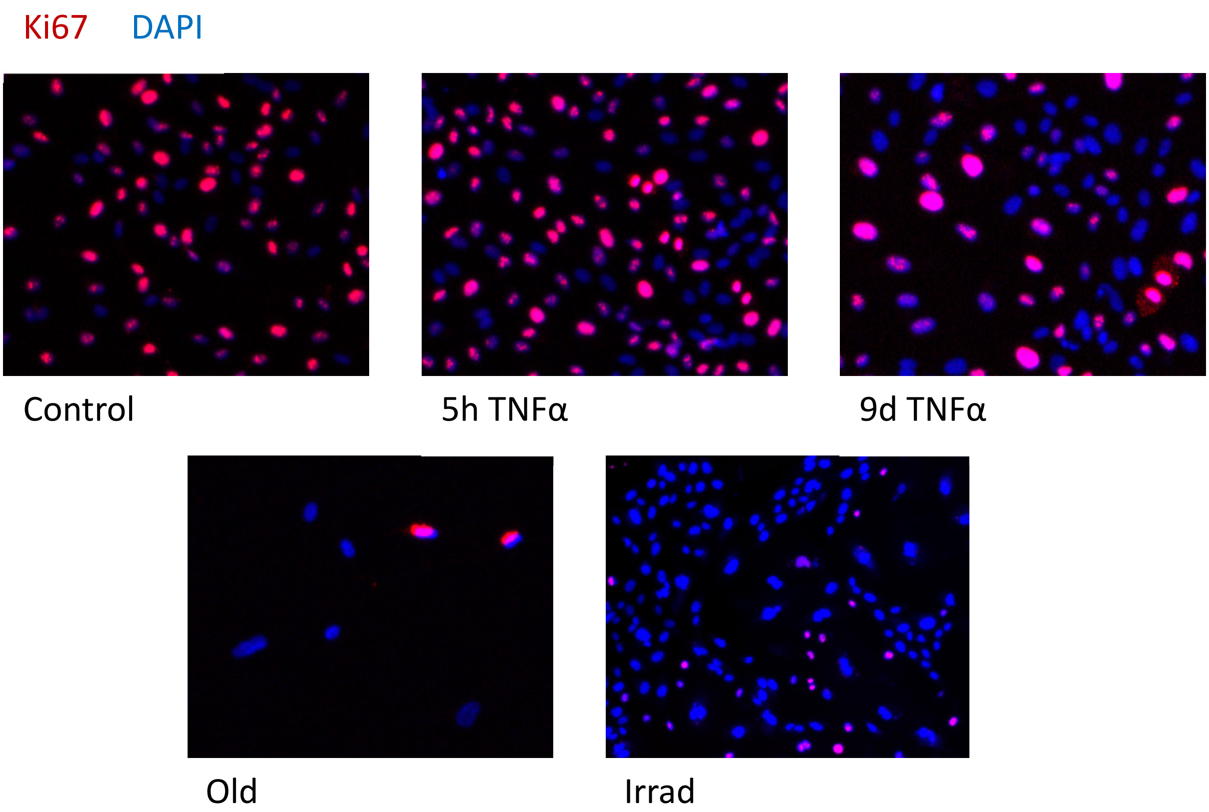

Supplementary Figure S2. Representative images of immunfluorecence staining of the proliferation marker Ki67 (in red), with Hoechst 33258 staining of nuclei (in blue). Quantification in the main text.

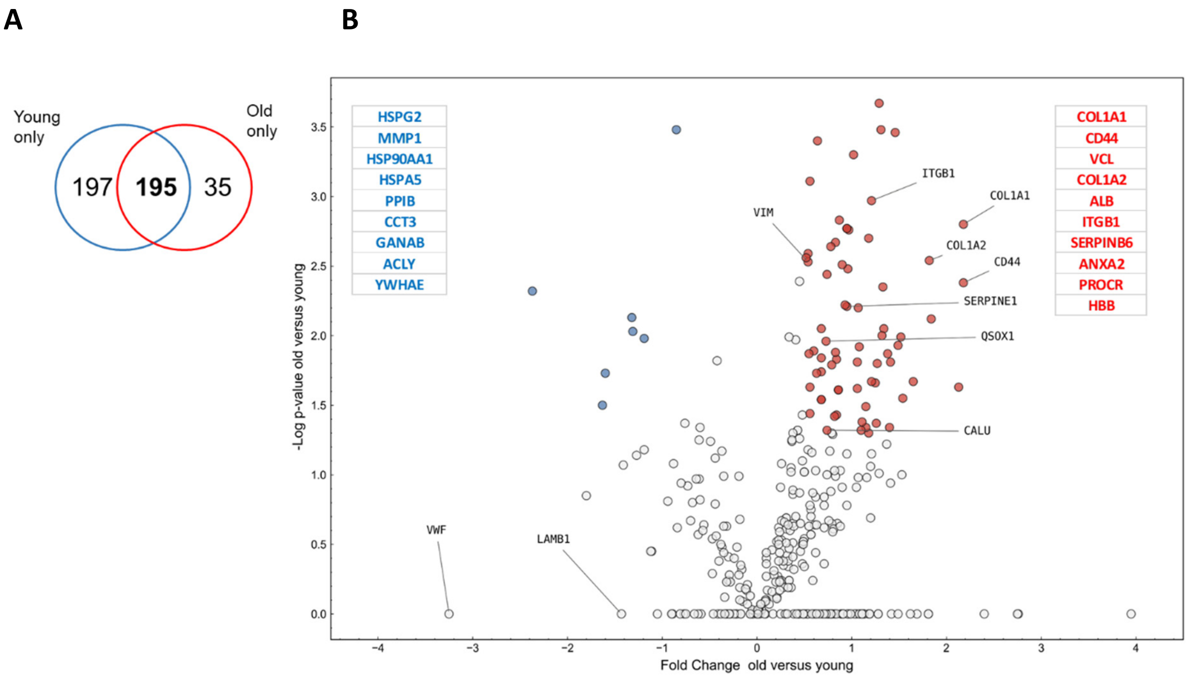

Supplementary Figure S3. Proteomic analysis of young and old HUVEC secretomes following short-term stimulation with PMA to promote secretion and EV release. (**A**) Venn diagram of unique and

common proteins identified after filtering for at least 3 valid values. (**B**) Volcano plot with significant regulated proteins (p.adj <0.05, fold change (FC) <-0.5 or >0.5. Top regulated proteins higher in young (blue) or in old HUVECs (red) are shown, proteins related to hemostasis and EMT are marked specifically.

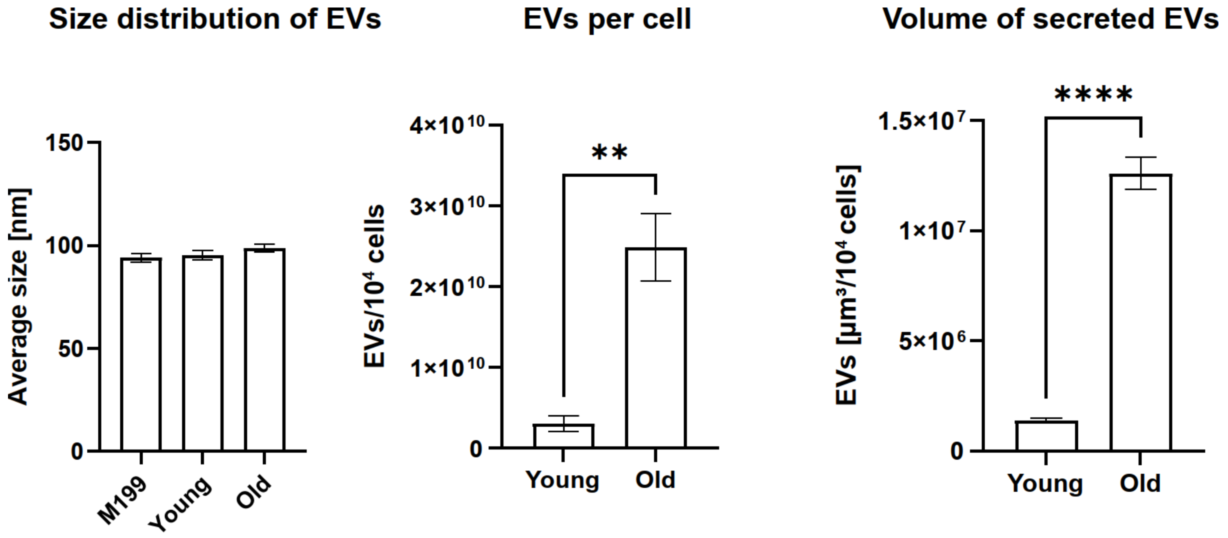

Supplementary Figure S4. Analysis of extracellular vesicles (EVs) in old endothelial cells (in replicative senescence) compared to young, proliferative cells. While the size distribution was very similar, the number of EVs released from the cells and their total volume was significantly higher in senescent cells as compared to the controls.

Supplementary Videos S1 – S4: show the time course of the scratch assay recorded on Incucyte equipment. S1: young HUVECs; S2: HUVECs treated for 5h with TNFα; S3: HUVECS treated for 9d with TNFα; S4: old HUVECs (in replicative senescence).

References for the supplementary materials:

1. Zhou, G.; Soufan, O.; Ewald, J.; Hancock, R.E.W.; Basu, N.; Xia, J. NetworkAnalyst 3.0: a visual analytics platform for comprehensive gene expression profiling and meta-analysis. *Nucleic Acids Res.* **2019**, *47*, W234–W241, doi:10.1093/nar/gkz240.

2. Shannon, P.; Markiel, A.; Ozier, O.; Baliga, N.S.; Wang, J.T.; Ramage, D.; Amin, N.; Schwikowski, B.; Ideker, T. Cytoscape: a software environment for integrated models of biomolecular interaction networks. *Genome Res.* **2003**, *13*, 2498–2504, doi:10.1101/gr.1239303.

3. Doncheva, N.T.; Morris, J.H.; Gorodkin, J.; Jensen, L.J. Cytoscape stringapp: network analysis and visualization of proteomics data. *J. Proteome Res.* **2019**, *18*, 623–632, doi:10.1021/acs.jproteome.8b00702.
